# Exploring the role of macrophages in the progression from atypical hyperplasia to endometrial carcinoma through single-cell transcriptomics and bulk transcriptomics analysis

**DOI:** 10.3389/fendo.2023.1198944

**Published:** 2023-09-14

**Authors:** Xiaolei Song, Re Na, Nianghai Peng, Wenming Cao, Yan Ke

**Affiliations:** Department of Gynecology, Shenzhen Hospital of Integrated Traditional Chinese and Western Medicine, Shenzhen, China

**Keywords:** ScRNA-seq, bulk-seq, macrophage, atypical endometrial hyperplasia, endometrial carcinoma

## Abstract

**Introduction:**

In this study, we aimed to identify key genes in endometrial cancer by conducting single-cell analysis of macrophages.

**Methods:**

We sourced clinical data from the TCGA database as well as supplementary datasets GSE201926 and GSE173682. Using bulk-seq data of atypical endometrial hyperplasia and endometrial cancer, we pinpointed key differentially expressed genes. Single-cell RNA sequencing was utilized for further gene expression analysis. Cluster analysis was conducted on TCGA tumor data, identifying two distinct subtypes. Statistical methods employed included LASSO regression for diagnostic modeling and various clustering algorithms for subtype identification.

**Results:**

We found that subtype B was closely related to cellular metabolism. A diagnostic model was established using LASSO regression and was based on the genes CDH18, H19, PAGE2B, PXDN, and THRB. This model effectively differentiated the prognosis of cervical cancer. We also constructed a prognosis model and a column chart based on these key genes.

**Discussion:**

Through CIBERSORT analysis, CDH18 and PAGE2B were found to be strongly associated with macrophage M0. We propose that these genes influence the transformation from atypical endometrial hyperplasia to endometrial cancer by affecting macrophage M0. In conclusion, these key genes may serve as therapeutic targets for endometrial cancer. A new endometrial cancer risk prognosis model and column chart have been constructed based on these genes, offering a reliable direction for future cervical cancer treatment.

## 1 Introduction

Atypical endometrial hyperplasia is a condition characterized by abnormal growth of the endometrium, specifically the glands. Pathologists observe cellular atypia in biopsy samples, which is a precursor to endometrial glandular carcinoma. The difference between atypical hyperplasia (benign growth) and non-atypical hyperplasia is the presence of cellular atypia. Endometrial hyperplasia is closely related to uterine tumors, with atypical adenoma and atypical hyperplasia being associated in 5.5% of patients, while atypical adenoma and endometrial carcinoma(UCEC) are associated in 5.9% of patients (1). Regarding treatment, for women with atypical endometrial hyperplasia or early-stage endometrial cancer, fertility-sparing treatment options include oral progestins or intrauterine devices, which have been extensively studied. These approaches are also recommended in the National Comprehensive Cancer Network (NCCN) guidelines for fertility-sparing treatment of endometrial cancer (2). However, the mechanism by which it progresses to endometrial carcinoma is not yet clear.

Endometrial carcinoma is a malignant tumor that occurs within the epithelium of the endometrium, and its incidence and disease-related mortality rates are gradually increasing worldwide (3). This type of cancer primarily affects postmenopausal women, with an average age of onset of 60 years old, and the likelihood of developing this disease is lower in women under 45 years old. Compared to Caucasian women, African-American women have a higher incidence and greater risk of death from this disease. Studies have found that the following factors are associated with an increased risk of endometrial carcinoma: nulliparity, late natural menopause (after age 55), estrogen-only hormone replacement therapy, hormonal therapy with tamoxifen for the treatment of breast cancer, and a family history of endometrial or colorectal cancer (4). The primary treatment for endometrial carcinoma is total hysterectomy with bilateral salpingo-oophorectomy. Radiation therapy and chemotherapy may also play a role in the treatment process (5). For women with highly malignant tumors, radiation therapy may be recommended after surgery. However, the problem of early diagnosis and poor treatment efficacy still exists, and there is a critical need to identify the oncogenic mechanisms and targets for precise treatment. Single-cell transcriptome sequencing (scRNA-seq) is an emerging sequencing technology that analyzes gene expression levels in individual cells within tissues, allowing for the study of cellular heterogeneity, differentiation of cell types and subtypes, identification of cell type-specific genes, and the discovery of cell dynamic processes. It has played a significant role in the study of cells and diseases (6).

In recent studies, single-cell transcriptomics have been successfully applied to investigate the development of endometrial cancer. For instance, one study utilized single-cell transcriptomics to analyze the function of macrophages in endometrial cancer, revealing their crucial role in the progression and metastasis of the tumor. Another study (reference: https://doi.org/10.3390/ijms23031237) disclosed the heterogeneity of macrophages in endometrial cancer, suggesting them as potential new targets for future treatments. These findings provide the background for our research, in which we aim to explore the role of macrophages in the transformation of atypical hyperplasia to endometrial cancer by analyzing single-cell and bulk transcriptomics data. Macrophages play an important role in endometrial cancer research. Macrophages are phagocytic cells of the innate immune system, including neutrophils, monocytes, and macrophages, which comprise approximately 25% of the immune environment in the endometrium. However, the phenotype and function of endometrial macrophages in health and disease are not fully defined, and studies have found that they are spatially and temporally regulated in tissue (7). There is a close relationship between macrophages and cancer. Macrophages are innate immune cells that play a critical role in tissue homeostasis, clearance of excess cells, and inflammation response to infection. Tumor-associated macrophages (TAMs) are key regulatory factors in the tumor microenvironment, and their complex interactions with the immune system and cancer are closely related. Studies have shown that in many cases, TAMs promote tumor progression rather than restrict tumor growth, which negatively impacts treatment response (8). In the early stages of tumor development, macrophages induce a pro-tumorigenic inflammatory environment that promotes tumor growth. As the tumor progresses to malignancy, macrophages induce angiogenesis, facilitate tumor cell migration and invasion, and suppress anti-tumor immunity. Macrophages are a key factor in unlocking the door to tumor cell escape, making it particularly important to study their involvement in the development of endometrial carcinoma.

## 2 Materials and methods

### 2.1 Data collection

Clinical information of endometrial cancer patients was collected from The Cancer Genome Atlas (TCGA) database (https://cancergenome.nih.gov/) (9), which included a total of 177 tumor samples and 24 normal samples. The information included in the study were gender, age, and stage. The GSE201926 dataset was downloaded from the Gene Expression Omnibus (GEO) database (10), which included 4 endometrial cancer samples and 8 atypical hyperplasia samples. scRNA-seq data was also downloaded from the GSE173682 dataset, which included samples from both diseases. Finally, copy number variation (CNV) data, somatic mutation data, and corresponding clinicopathological information of endometrial cancer were retrieved from the Cancer Genome Atlas (https://portal.gdc.cancer) (11).

### 2.2 Single-cell analysis

Further analysis was performed on the single-cell (12) dataset GSE173682, which included 2 endometrial cancer samples. For each batch of cells, we calculated the number of genes expressed by each cell. Genes with expression levels lower than 0.1% of the cell count were excluded from the study. The batch of cells did not contain mitochondrial genes. Ultimately, all cells in the dataset met quality control standards. Principal component analysis (PCA) (13)was performed using the Seurat package (14) (version 3.2.2) in R software (version 4.0.2), along with JackStraw and PCEIbowPlot (15) functions, to select important principal components (PCs). Seurat’s FindAllMarkers function was used to identify specific genes for each cell subgroup. The RunUMAP (16) function was then used for cell clustering and visual analysis using UMAP. Marker genes were subsequently annotated using the singleR package (17) and corrected using CellMarker based on their characteristics. The Seurat package (version 3.2.3) and DoubletFinder package (18) (version 2.0.3) were used in R (version 3.6.3) to filter cells and genes. Only cells that met the following quality control standards were retained for further data analysis: (i) the number of detected genes was less than two and a half times the average number of expressed genes in cells from the same sample; (ii) mitochondrial gene expression was less than 20% of the total count in a cell; and (iii) DoubletFinder’s standard workflow was used to remove doublets. The samples were merged into one object, and Harmony software package (19)(version 1.0) was used for unsupervised clustering after filtering cells. Genes were retained only when expressed in at least 10 cells. Differentiated expression genes (DEG) in each cluster were identified using the Seurat function FindMarkers, and only the macrophage clusters were annotated. The data was divided into macrophage clusters and non-macrophage clusters.

### 2.3 GEO differential analysis and identification of key genes

We then performed differential analysis on the endometrial hyperplasia group and endometrial cancer group in the GSE201926 dataset using the limma package (20), with a screening criterion of p<0.05. Key genes related to macrophages were screened from the single-cell dataset, with screening criteria of min.pct of 0.05 and logfc.threshold of 0.05. The intersection of these two gene sets was obtained as the key genes for subsequent analysis.

### 2.4 Consensus clustering analysis of key genes

Following the identification of key genes from the previous step, all genes were found to be expressed in TCGA. The LASSO (21) algorithm was used to evaluate the prognostic value of these key genes. Based on the LASSO regression results, immune cells that were correlated with prognosis were selected for further validation through survival analysis. The “network” package was used to construct a gene network for the key genes. ConsensusClusterPlus R package (22) was used for unsupervised clustering analysis to generate different clustering subtypes based on the expression of apoptosis macrophage genes using the K-Means algorithm (23). After clustering, the sample size of each cluster was not small. The clustering had high intra-cluster similarity and low inter-cluster similarity. Gene Set Variation Analysis (GSVA) was performed on gene sets compiled from MSigDB (24) (C2.Cp.ke.v7.2) to discover biological differences between molecular subtypes. The tumor microenvironment of each subtype was also analyzed.

### 2.5 Development of prognosis model

Using the identified key genes, uterine endometrial cancer patients were divided into two subgroups (high-risk and low-risk). The TCGA data was randomly divided into train and test sets in a 7:3 ratio. To avoid the influence of random allocation bias on the stability of subsequent modeling, all samples were returned to the random allocation 200 times, and the samples were grouped and sampled in a 1:1 ratio according to the training and validation sets. Based on the median risk score of the train set, all samples were divided into high-risk and low-risk subgroups, and survival analysis and ROC (25) curve were performed on both groups. The PCA (principal component analysis) and t-SNE (t-distributed stochastic neighbor embedding) analyses were also performed using the “ggplot2” R package (26).

### 2.6 Development of prediction nomogram

Using the risk score and clinical features of uterine endometrial cancer patients, a prediction nomogram was created using the “nomoR” package (27). Each variable had a score, and the total score was obtained by adding up the scores for each sample. A calibration plot was used to depict the predicted values of survival events at 1, 3, and 5 years compared to the virtual observed values. Time-dependent ROC curves were used to evaluate the nomogram for 1-, 3-, and 5-year survival rates.

### 2.7 Immune cell infiltration correlation analysis and drug sensitivity analysis

The CIBERSORT algorithm (28) was then used to estimate the proportions of immune cell infiltration for each uterine endometrial cancer sample, using cell-specific gene features to distinguish 22 immune cell groups. The “PERM” parameter was set to 1000 and the cutoff value was set to p < 0.05. In addition, the proportion of each immune cell in the sample was calculated, and a bar graph was used to display this information. The “pheatmap” package (29) was used to create a heatmap of the 22 immune cells, and the “vioplot” package was used to display their abundance. The “corrplot” package was used to create a correlation heatmap to visualize the correlation between the 22 different immune cells. Furthermore, the correlation between immune cell levels and key genes was also analyzed. The “pRRophetic” package (30) was used to predict the sensitivity of uterine endometrial cancer to certain drugs, and boxplots of the inhibitory effect of drugs on cancer cells were drawn using the “ggboxplot()” and “ggplot2” packages.

### 2.8 Tumor mutation analysis

In our study, in addition to the samples previously described, we also selected 504 tumor mutation samples from The Cancer Genome Atlas (TCGA) database for analysis. Among these 504 samples, 82 had mutations. Tumor mutation analysis is based on sequencing data from tumor and normal samples, identifying mutation events that occur specifically in tumor samples by comparing the differences between the two samples. Generally, tumor mutation analysis includes various analyses such as single nucleotide variations (SNV), small fragment insertions/deletions (indel), and copy number variations (CNV). We used the SNV and CNV data of endometrial cancer and the R package maftools for analysis. Maftools is an R package (31) specifically designed for analyzing mutation annotation format (MAF) files, which can help users obtain various biological information and analysis results from MAF files and visualize these results. The plotCNA() function was used to generate a CNV spectrum plot, which shows the copy number variation of genes. The CNV spectrum plot provides information on the position, gene, and copy number of the copy number variation. This separate set of samples was selected to enhance the statistical power and provide a more extensive understanding of the mutation landscape in endometrial cancer, enabling a deeper insight into the genetic alterations that drive the disease.

## 3 Results

### 3.1 Analysis of differentially expressed genes

**Figure 1:**
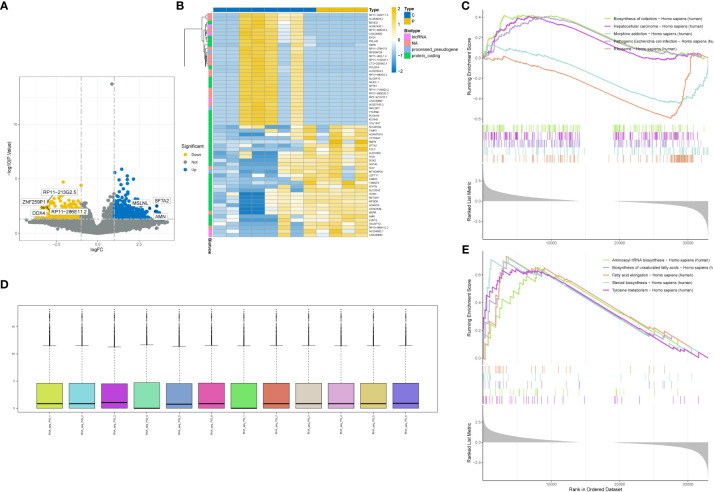
Differential gene analysis. **(A)** Volcano plot showing differentially expressed genes. **(B)** Heatmap showing differentially expressed genes. **(C)** GSEA enrichment of downregulated genes in the gene set with the highest enrichment score. **(D)** Box plot showing data normalization. **(E)** GSEA enrichment of upregulated genes in the gene set with the highest enrichment score.

We performed differential analysis on the standardized GSE201926 dataset and obtained 2250 differentially expressed genes. The distribution of the standardized data is shown in **Figure 1D**. We visualized the differentially expressed genes using a volcano plot (**Figure 1A**) and a heat map (**Figure 1B**), and performed GSEA enrichment analysis on the differentially expressed genes. **Figure 1C** shows the enriched pathways for downregulated genes, including Biosynthesis of cofactors, Hepatocellular carcinoma, and Morphine addiction. **Figure 1E** shows the enriched pathways for upregulated genes, including Aminoacyl-tRNA biosynthesis, Biosynthesis of unsaturated fatty acids, and Steroid biosynthesis.

### 3.2 Single-cell analysis results

**Figure 2:**
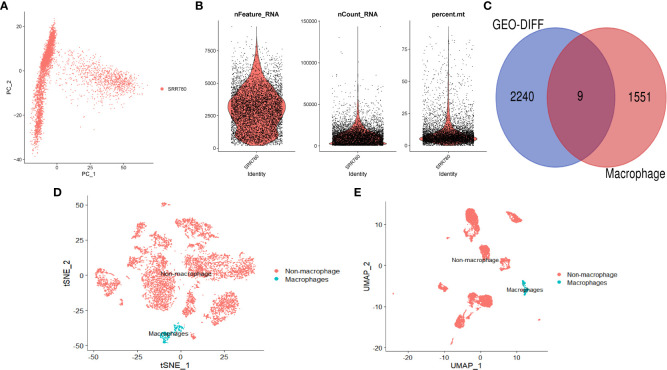
Single-cell analysis. **(A)** PCA dimension reduction plot of the data. **(B)** Data feature plot showing the number of genes detected per single cell (“nfeature”), frequency (“ncount”), and percentage (“percent”). **(C)** Venn diagram showing the intersection of differentially expressed genes and macrophage-related genes. **(D)** Annotation results of single-cell data based on tSNE. **(E)** Annotation results of single-cell data based on UMAP.

We performed further analysis on the single-cell dataset (GSE201926)and used PCA to reduce the high-dimensional data to low-dimensional data, which was then visualized (**Figures 2A, B**). We used the FeaturePlot function to annotate the data and visualize the macrophages using three marker genes (CD163, CD14, and CSF1R), as shown in **Figures 2D** (tSNE) and **2E** (UMAP). We then filtered the macrophage data and obtained 1559 genes. We intersected the differentially expressed genes and macrophage-related factors and obtained 9 key genes for further analysis, including GINS3, PRMT2, MERTK, WWP2, NEK6, NACC1, NR1H3, ATP2A2, and TM4SF19. These 9 genes were visualized using a Venn diagram (**Figure 2C**).

### 3.3 Cluster analysis

**Figure 3:**
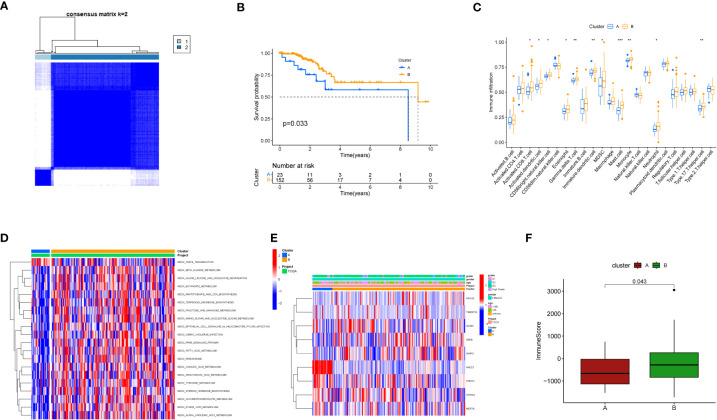
Clustering analysis based on key genes. **(A)** Consensus matrix heatmap defining two clusters and related regions. **(B)** Kaplan-Meier curves of OS for the two gene subtypes (p<0.05). **(C)** Abundance of 22 immune cell infiltrations in the two subtypes. The asterisk represents statistical P values (*P < 0.05; **P < 0.01; ***P <0.001). **(D)** GSVA of biological pathways between different subtypes. The heatmap is used to visualize these biological processes, with red indicating activated pathways and blue indicating inhibited pathways. **(E)** Relationship between the two subtypes and clinical pathological features. **(F)** Differences in immune scores between the two clusters in the direct immune microenvironment.

Further, we employed a consensus clustering algorithm to categorize TCGA cancer patients based on the expression profile of the aforementioned 9 key genes. According to our findings, k=2 was identified as the optimal clustering variable to divide the dataset into Cluster A and Cluster B (**Figure 3**A). Based on survival analysis, these clusters could distinguish prognostic status well with p=0.033 (**Figure 3B**). According to immune infiltration results, differences were found between these two clusters across multiple immune cells (**Figure 3C**), such as CD4 T cells, MDSC cells, and Monocyte cells. We also conducted GSEA enrichment analysis (**Figure 3D**) and found that Cluster B was more enriched in multiple pathways than Cluster A, such as KEGG_BUTANOATE_METABOLISM and KEGG_TERPENOID_BACKBONE_BIOSYNTHESIS. Additionally, a correlation heatmap was drawn based on these 9 key genes (**Figure 3E**). In an analysis focusing on the direct immune microenvironment, we observed distinct differences in immune scores between the two clusters, labeled as Cluster A and Cluster B. The variations were statistically significant, with a p-value of 0.043, suggesting that the immune response in these two clusters is notably different (**Figure 3F**).

### 3.4 Mutation analysis

**Figure 4:**
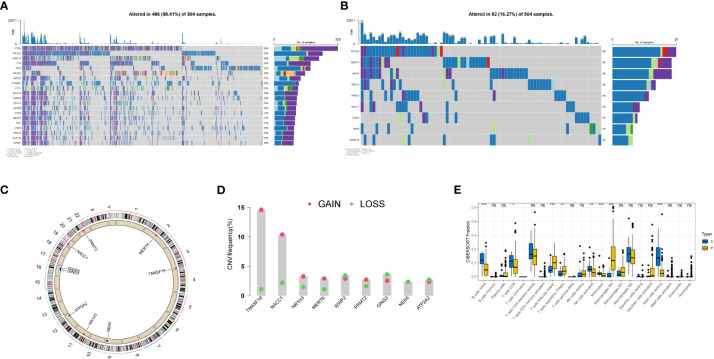
Genetic variation landscape of key genes in endometrial cancer. **(A)** Waterfall plot of SNV mutations, with the numbers on the right indicating the mutation frequency of each gene. The bar chart on the right shows the proportion of mutations. The stacked bar chart below shows the proportion of transformations. The results of the top 20 genes mutated in endometrial cancer are shown. **(B)** Waterfall plot of SNC mutations showing the mutation results of selected key genes in endometrial cancer. **(C)** The positions of CNV in key genes on 23 chromosomes. **(D)** The frequency of CNV gains and losses in key genes. The column represents the frequency of changes, with red dots representing gain frequency and green dots representing loss frequency. **(E)** Immune infiltration analysis in TCGA transcriptome data, with blue representing normal samples and yellow representing tumor samples. * represents less than 0.05, ** represents less than 0.01, *** represents less than 0.001.

We plotted waterfall diagrams for the top 20 mutated genes in the tumor (**Figure 4A**) and for the 9 selected genes (**Figure 4B**). Among the 504 samples which from TCGA SNV database, 82 had mutations, resulting in a mutation rate of 16.27%. The highest mutation rate was for ATP2A2 (6%), followed by MERTK and WWP2 (5%). We also plotted the distribution of these genes on the chromosomes (**Figures 4C, D**) and found that the GAIN of TM4SF19 was significantly higher than the LOSS. We further analyzed the immune infiltration in the tumor using TCGA data and found that the macrophages (M0) were significantly present, indicating a potential role in the tumor environment. In the immune infiltration analysis conducted on TCGA transcriptome data, we utilized blue to represent normal samples and yellow for tumor samples. We observed significant differences in multiple immune cell types between the normal and disease groups. Notably, distinct variations were found in B cells native, T cells follicular helper, and Monocytes among others (**Figure 4E**).

### 3.5 Modeling analysis

**Figure 5:**
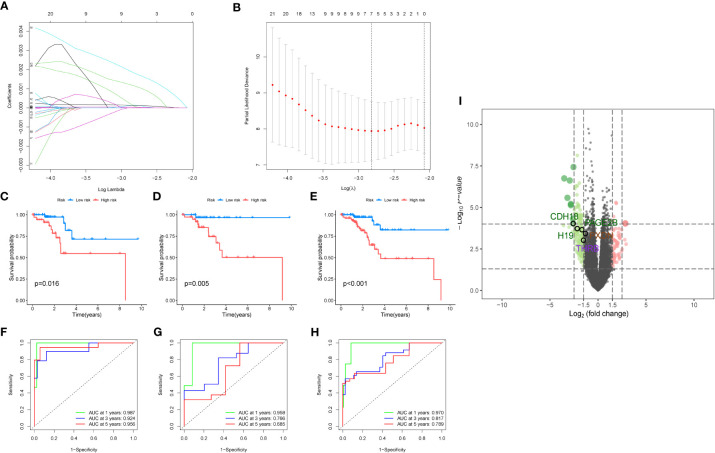
Modeling analysis results. **(A)** Lasso regression analysis results. The figure shows the sparsity and predictive performance of the Lasso regression model at different l values. As the l value increases, the sparsity of the model increases, meaning that there are fewer parameters in the model. **(B)** The figure is used to select the optimal regularization coefficient l to obtain a Lasso regression model that balances sparsity and predictive performance. **(C–E)**. Survival analysis results for the overall data, training group, and test group, respectively. **(F–H)**. Survival analysis results for the overall data, training group, and test group, respectively. **(I)** The volcano plot depicts the differential analysis between the two clusters.

Then we performed differential analysis between the two clusters and selected using TCGA cancer database, which differentially expressed genes with logfc>1 and p-value<0.05. Then, we conducted a multivariate Cox analysis to further screen the genes with p<0.05, and obtained 65 key genes. These genes were used for lasso analysis to construct the model, as shown in **Figure 5A**, which displays how the coefficients of each feature change at different penalty strengths. By adjusting the value of lambda, the complexity of the model can be controlled to better fit the data. When lambda approaches 0, all the coefficients of the features are non-zero, indicating the highest complexity of the model. When lambda increases, some feature coefficients become zero, which means that these features are considered irrelevant or unimportant, making the model simpler. **Figure 5B** shows that at a certain lambda value, the performance of the model will reach its best level, and the value of Partial Likelihood Deviance will be minimized. Therefore, this figure can be used to select the appropriate lambda value to obtain the best performance of the Lasso model. Feature selection is performed using lasso regression (Least Absolute Shrinkage and Selection Operator), applied to the COX proportional hazard model for survival analysis. Cross-validation is employed to select the appropriate lambda value (i.e., regularization parameter), allowing the model to achieve optimal performance. Key genes screened out from the lasso regression are used to build a COX proportional hazard model. Based on the median of the risk scores, patients are classified into two groups: high-risk and low-risk. We randomly divided the TCGA data into a train group and a test group at a ratio of 7:3, and constructed a risk model using lasso screening with 5 genes, namely CDH18, H19, PAGE2B, PXDN, and THRB. The survival curves of the total group, train group, and test group are shown in **Figures 5C–E**, with p<0.05. The corresponding ROC curves are displayed in **Figures 5F–G**, with the ROC curves of the total group at 1, 3, and 5 years being 0.987, 0.924, and 0.956, respectively; the ROC curves of the train group being 0.970, 0.817, and 0.789, respectively; and the ROC curves of the test group being 0.958, 0.766, and 0.685, respectively. The volcano plot of differential analysis between the two clusters is shown in **Figure 5I**.

**Figure 6:**
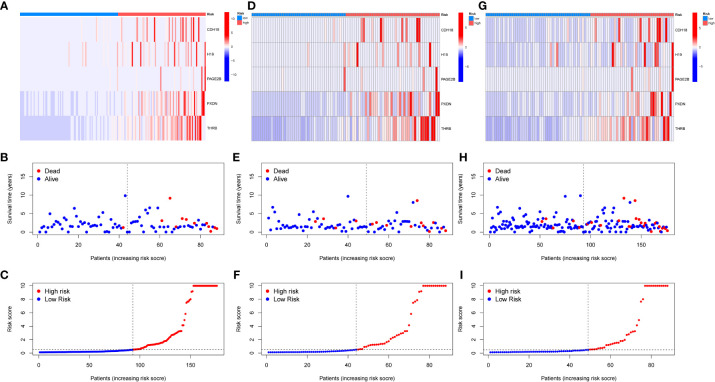
Prognosis of risk model in training and testing groups. **(A–C)**. Overall results. **(D–F)**. Results for the training group. **(G–I)**. Results for the testing group. **(B, E, H)** show the survival time and survival status between the low-risk group and the high-risk group in the whole group, training group, and testing group, respectively. **(A, D, G)** show the expression heatmap of key genes in different risk groups in the whole group, training group, and testing group, respectively.

A diagnostic model was constructed based on the 5 selected key genes, and combined with clinical data to classify endometrial cancer patients into high-risk and low-risk groups using the risk score formula. Patients with high-risk scores had a higher mortality rate than those with low-risk scores. The analysis results of the total group are shown in **Figures 6A–C**, the train group analysis results are shown in **Figures 6D–F**, and the test group analysis results are shown in **Figures 6G–I**. According to the constructed model, heat maps in **Figures 6A, D**, **E** show the expression profiles of risk genes mRNA. **Figures 6C, 6I** show that the expression of risk type mRNA was significantly upregulated with the increase of risk score in endometrial cancer patients.

### 3.6 Immune correlation analysis

**Figure 7:**
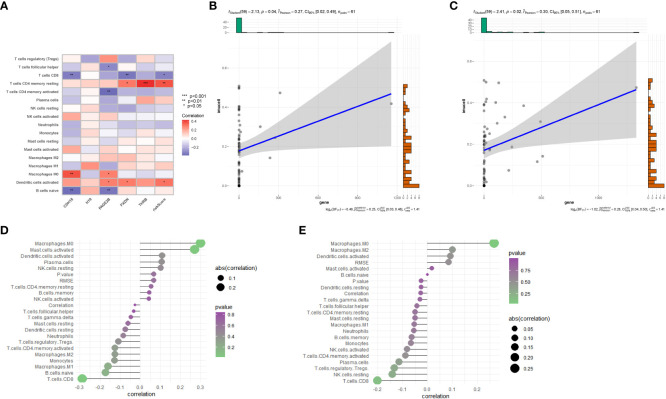
Immune-related analysis. **(A)** Correlation analysis between immune cell infiltration results in the prognostic model and five genes. **(B)** Correlation between PAGE2B and M0 macrophages. **(C)** Correlation between CDH18 and M0 macrophages. **(D)** Correlation analysis of CDH18 with various cells. **(E)** Correlation analysis between PAGE2B and various cells..

Further utilizing CIBERSORT, we associated the five key genes(CDH18,H19,PAGE2B,PXDN and THRB) with immune cell infiltration to explore their correlation with immune cells. **Figure 7A** shows the correlation of risk genes with various tumors, and we found that CDH18 and PAGE2B are significantly positively correlated with macrophage M0 cells, with a p-value less than 0.05. Furthermore, we displayed the correlation maps of these two key genes with various cells in **Figures 7D, E**, respectively. **Figure 7B** shows the correlation of PAGE2B with macrophage M0 cells with a cor value of 0.27 and a p-value of 0.04, while **Figure 7C** shows the correlation of CDH18 with macrophage M0 cells with a cor value of 0.3 and a p-value of 0.02.

**Figure 8:**
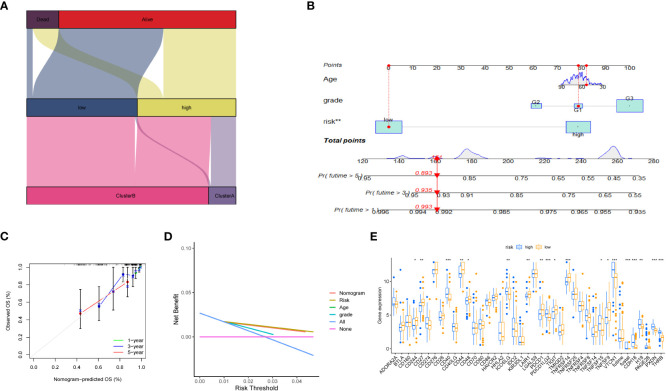
Volcano plot and immune analysis. **(A)** Sankey diagram based on clustering and modeling analysis in bioinformatics. **(B)** Construction and evaluation of the Nomogram based on key genes. The Nomogram is used to predict the 1-year, 3-year, and 5-year OS of EC patients. **(C)** Calibration curve of the column chart. **(D)** Kaplan-Meier curve for immune infiltration analysis. **(E)** The expression distribution of immune-related genes in different risk groups. * represents less than 0.05, ** represents less than 0.01, *** represents less than 0.001.

Furthermore, we depicted the correlation between the clustering subtype and the risk model in **Figure 8A**. We estimated the OS of endometrial cancer patients for clinical application based on the association between the risk score and patient prognosis and created a column chart containing clinical features (**Figure 8B**). Overall Survival is defined as the duration of time from either the date of diagnosis or the start of treatment for a disease, such as cancer, that a patient is still alive. Based on this column chart, we estimated the 1-, 3-, and 5-year OS of patients (**Figure 8C**). Calibration curves demonstrate the actual observed and predicted parameters (**Figure 8D**). Additionally, we compared the predictive accuracy of the column chart with other clinical variables, and the results indicated that the column chart has better survival prediction. To analyze the ability of the risk score to predict potential checkpoint blockade therapy, we plotted a box plot to show the difference in immune checkpoint gene expression between the high- and low-risk groups (**Figure 8E**).

### 3.7 Tumor microenvironment and drug sensitivity subtype

**Figure 9:**
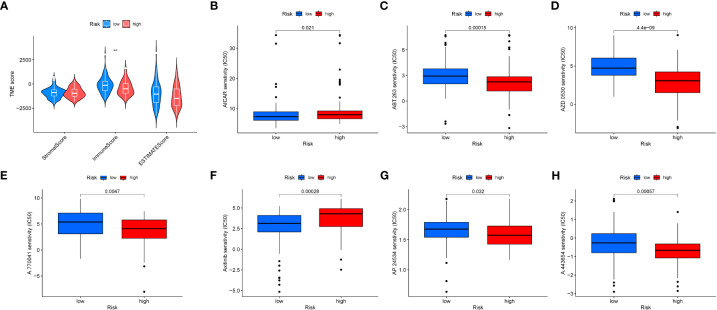
Tumor microenvironment and drug sensitivity analysis. **(A)** Box plot showing the expression differences of the tumor microenvironment in different risk groups. **(B–H)**. Drug sensitivity analysis in different risk groups.

We evaluated the relationship between immune cell abundance and prognostic features, and calculated the Tumor Microenvironment (TME) score for the two risk groups. Immune cell infiltration includes various immune cell types, such as T cells, B cells, natural killer cells, and the previously mentioned macrophages (M0). We found that the immune score was higher in the low-risk group (**Figure 9A**), indicating that tumors in the low-risk group have more immune cell infiltration, which may signal better prognosis and treatment response. Additionally, six drugs showed significant differences in the half-maximal inhibitory concentration (IC50) values between the high- and low-risk groups (**Figures 9B–H**), including AICAR (Aminoimidazole Carboxamide Ribonucleotide activator), ABT.263 (Bcl-2 family protein inhibitor), AZD.0530 (Src/Abl inhibitor), A.770041 (Lck inhibitor), Axitinib (multitargeted tyrosine kinase inhibitor), AP.24534 (BCR-ABL inhibitor), and A.443654 (Akt inhibitor).

## 4 Discussion

With a full understanding of endometrial cancer as the most common gynecologic malignancy, and taking into consideration the standard treatment approaches outlined in the introduction, our study focuses on the role of macrophages in endometrial cancer and specific macrophage genes. We explored how macrophages shield lesions from immune surveillance while promoting pathological cell growth, invasion, and metastasis (32), and identified 9 key macrophage genes, including GINS3, PRMT2, MERTK, WWP2, NEK6, NACC1, NR1H3, ATP2A2, and TM4SF19, some of which have not been previously reported in the context of endometrial cancer. In endometriosis and cancer of the endometrium, macrophages can protect lesions from immune surveillance while promoting pathological cell growth, invasion, and metastasis (32). The study also found that the density of CD68-positive macrophages in endometrial cancer tissue is higher than that in normal endometrial tissue. It is worth noting that macrophages appear to support the role of estrogen and progesterone in endometrial cancer. Jiang et al. found that the increase in macrophages in endometrial cancer is associated with a decrease in progesterone receptor expression (32). In our study, a total of 9 key macrophage genes were identified, including GINS3, PRMT2, MERTK, WWP2, NEK6, NACC1, NR1H3, ATP2A2, and TM4SF19. Previous studies have suggested that GINS3 may play a role in endometrial cancer (33). MERTK is a promising therapeutic target on tumor-associated macrophages in most solid cancers, and its ligands may be attractive molecular targets for treating endometrial cancer. As multifunctional suppressors of immune cells, they efficiently regulate phagocytic clearance of apoptotic cells and make the tumor microenvironment more conducive to tumor growth, which is consistent with our findings (34). However, there have been no reports on the involvement of PRMT2, NEK6, NACC1, ATP2A2, and TM4SF19 in endometrial cancer. On the other hand, it has been found that loss of Prmt2 function can alter the nuclear accumulation of NF-κB in stimulated macrophages (35) Furthermore, PRMT2 overexpression can inhibit the formation of RAW 264.7 macrophage foam cells induced by oxidized low-density lipoprotein (36). It is closely related to macrophages. On the other hand, our GSEA analysis of differential genes between atypical hyperplasia and endometrial cancer showed that the upregulated genes in the gene set with the highest enrichment score were mainly enriched in metabolic pathways in the organism, such as aminoacyl-tRNA biosynthesis, biosynthesis of unsaturated fatty acids, and steroid biosynthesis. At the same time, when we performed cluster analysis on these nine key genes, we also found the same conclusion that subtype B patients had better OS, and these pathways mainly involved various aspects of cellular metabolism, including the synthesis, degradation, and utilization of glucose, fatty acids, amino acids, and nucleotides. For example, KEGG_BETA_ALANINE_METABOLISM, KEGG_VALINE_LEUCINE_AND_ISOLEUCINE_DEGRADATION,KEGG_BUTANOATE_METABOLISM,andKEGG_FRUCTOSE_AND_MANNOSE_METABOLISM are all metabolic pathways, indicating that they are closely related to metabolism. We believe that these key factors affect the transition from atypical hyperplasia to endometrial cancer by influencing metabolic pathways in macrophages. In a related context, the role of NR1H3, encoding for the LXR-α isoform, in macrophages has been explored in diffuse large B-cell lymphomas (DLBCL). By regulating lipid metabolism, NR1H3 controls macrophage polarization and the inflammatory response, thereby influencing the tumor microenvironment (TME) (37). In a detailed study, high expression of the NR1H3 receptor transcript was identified in M1-like pro-inflammatory macrophages, suggesting a critical role in DLBCL progression. Remarkably, NR1H3^high patients displayed longer survival compared to NR1H3^low cases, indicating the gene’s prognostic potential. This finding prompts future investigations into its therapeutic applications and provides insights that may extend to macrophage-mediated development in other cancer types, including endometrial cancer. We further explored these hypotheses and found through immune infiltration that in tumor tissues, the M0 macrophages were significantly higher than in normal tissues. Previous studies have shown that the numbers of M0 and M1 macrophages are higher in cancer tissues than in normal tissues. In addition, the study found that as the stage of endometrial cancer increases, the levels of activated M0 and CD8+ T cells decrease, while the levels of M1 and M2 macrophages increase (38). Comparing our results with recent findings using similar approaches on the role of macrophages in the progression of hyperplasias into carcinomas, we observe intriguing parallels and distinctions. Recent studies highlight how macrophages can contribute to the progression of hyperplasias into malignancies by influencing specific pathways and modulating the immune response within the tumor microenvironment. The insights gained from these comparisons not only reinforce our understanding of the macrophages’ role in endometrial cancer but also provide critical directions for further exploration. Our identification of key macrophage genes aligns with other research, expanding our comprehension of the complex interaction between immune cells and cancer development (39). Moreover, in the majority of cancer types, including those of the breast and lung, the presence of M0 macrophages has been consistently linked to a poor prognosis (38). These observations align with our findings. In further analyses, we focused on differentially expressed genes within this cluster, selecting those that displayed the most pronounced changes for subsequent model analysis. **Figures 7B, C** demonstrate significant correlations between two genes, PAGE2B and CDH18, with macrophage M0 cells in endometrial cancer tissues. Specifically, PAGE2B correlates with macrophage M0 cells with a correlation value (cor) of 0.27 and a p-value of 0.04, while CDH18 shows a correlation with macrophage M0 cells with a cor value of 0.3 and a p-value of 0.02. These correlations suggest that PAGE2B and CDH18 may play essential roles in macrophage-mediated development of endometrial cancer, possibly influencing the immune response and tumor microenvironment. While there is no previous report directly connecting CDH18 with macrophages, this finding is consistent with studies highlighting the role of specific genes in macrophage function and their potential impact on tumor development (39). PAGE2B is a coding unit associated with tumor-associated antigens. It is a tumor-specific antigen and is highly expressed in tumor tissues (40).There is no previous report on the correlation between CDH18 and macrophages. We speculate that it could be a target gene for therapy, and its influence on the transition from atypical hyperplasia to endometrial cancer could be through M0 macrophages. The correlation of CDH18 with macrophage M0 cells may indicate its significant role in tumor differentiation and prognosis in endometrial cancer. CDH18, a member of the cadherin superfamily, is known to be involved in cell-cell adhesion, cell signaling, and tissue organization. Its correlation with M0 macrophages in our study could imply a functional relationship in shaping the tumor microenvironment, influencing immune responses, and potentially affecting the progression and differentiation of the tumor. Although there have been limited studies on CDH18 in the context of endometrial cancer, its connection with macrophages presents an intriguing direction for further exploration. Investigating CDH18’s expression, function, and regulation within the tumor might reveal novel insights into endometrial cancer differentiation, providing valuable prognostic information and possibly unveiling new therapeutic targets.

The innovation of our study lies in the combination of the impact of macrophage-related genes on the transition from atypical hyperplasia to endometrial cancer. In addition to developing key gene-related features and heat maps, we have comprehensively revealed a gene prediction model for endometrial cancer based on key genes in macrophages and validated it in the TCGA dataset, providing a promising macrophage-related target for endometrial cancer patients. These results suggest that the identified key genes are promising potential targets for the development of treatment strategies. The limitations of this study include a limited sample size, a focus on specific cancer subtypes, and certain constraints in the assumptions made in our analysis. A broader and more diverse sample would help further validate our findings. However, these results were obtained from analysis of public databases, and there may not be significant variations in clinical practice. Therefore, more clinical patients are needed to confirm our conclusions. Our research results provide new ideas and directions for improving the clinical effectiveness of immunotherapy, identifying different endometrial cancer immune phenotypes, and promoting future precise and personalized immunotherapy for endometrial cancer patients.

## Data availability statement

The datasets presented in this study can be found in online repositories. The names of the repository/repositories and accession number(s) can be found in the article/supplementary material.

## Author contributions

XS and RN were instrumental in the conceptualization and design of the research, as well as in the acquisition, analysis, and interpretation of the data. XS is mainly responsible for the data analysis part. NP assisted in data interpretation and statistical analysis. WC involved in the project administration and supervision helped in the initial drafting of the manuscript and provided critical revisions. YK provided substantial contributions to the writing and critical revising of the manuscript. All authors read and approved the final manuscript.
